# Coupling and Preload Analysis of Piezoelectric Actuator and Nonlinear Stiffness Mechanism

**DOI:** 10.3390/mi16091024

**Published:** 2025-09-06

**Authors:** Wei Wang, Jinchuan Zheng, Zhe Sun, Xiaoqi Chen

**Affiliations:** 1 School of Engineering, Swinburne University of Technology, Melbourne, VIC 3122, Australia; 2 College of Information Engineering, Zhejiang University of Technology, Hangzhou 310023, China; 3 School of Intelligent Engineering, South China University of Technology, Guangzhou 511442, China

**Keywords:** piezoelectric actuators, compliant mechanisms, preload effects, variable-stiffness mechanisms, precision positioning systems

## Abstract

This article presents a comprehensive investigation of the dynamic coupling between a piezoelectric actuator (PZT) and its driving nonlinear stiffness mechanism (NSM) stage for precise positioning control. Particular emphasis is placed on the preload-induced effects on the force transmission and structural separation between the PZT and NSM. To ensure continuous mechanical contact between them, we propose a no-separation criterion based on acceleration matching, from which the minimum preload requirement is analytically derived. Additionally, static and dynamic simulations reveal that increasing the preload force from 0 N to 10 N can push the first natural frequency of the holistic system from 214.21 Hz to 258.17 Hz, respectively. This beneficially enhances the displacement consistency across different geometric configurations. Moreover, an appropriate preload force can prevent separation and increase system stiffness while reducing nonlinear deformation. Experimental results verifies that a preload of 10 N can prevent the separation between the PZT and NSM stage and maintain achievable output displacement of the stage within the range from 54.35μm to 129.42μm. This article offers the analytical results of preload setting to guarantee reliable actuation for nonlinear precision positioning stages.

## 1. Introduction

Precision positioning systems are widely used in advanced technologies that require nanoscale accuracy and fast dynamic response [1,2]. Common applications include optical alignment, micro/nano-fabrication, atomic force microscopy, and biomedical instruments. These systems often operate in confined spaces where structural compactness, high resolution, and fast actuation must be achieved simultaneously [3].

Among the available actuators, piezoelectric actuators (PZTs) are widely used due to their high force output, sub-nanometer resolution, fast response, and simple structure [4,5,6,7,8]. These advantages make PZT suitable for precision motion control. However, the stroke of PZT is very small usually only a few tens of micrometers [9], which limits their use in systems requiring large displacements. To address this limitation, displacement amplification mechanisms are commonly used to extend the motion range [10,11].

Compliant mechanisms are widely used for amplification because they use elastic deformation to transfer motion without mechanical joints, friction, or backlash [12]. Their single-piece structure improves reliability, reduces system size, and offers flexibility in design [13]. In addition, their geometry can be tailored to the actuator output and provide suitable amplification ratios [14]. This approach helps maintain compactness while achieving large motion and stable dynamic behavior. As a result, compliant mechanisms are often integrated with PZTs in high-precision, large-stroke stages [15,16].

Despite these benefits, integrating PZT with compliant mechanisms introduces bidirectional coupling. The compliant structure alters the actuator boundary conditions, affecting its stiffness and motion. At the same time, the force and stiffness from the actuator influence how the structure deforms and transfers the force [17,18,19]. This interaction becomes critical in compact systems where small changes in preload or external load can result in performance degradation.

Preload is often applied to eliminate mechanical gaps and maintain contact between the PZT and the compliant mechanism during operation [19]. However, it also introduces internal stress into the system, which may reduce actuator stroke, particularly when small PZT is used [20]. In actuators with larger stroke capacity, this effect is often negligible; but in miniaturized systems, it must be carefully considered [11,18,21].

Maintaining continuous contact is also essential during dynamic motions. Even brief and tiny separation can cause uncontrolled movement, loss of actuation force, or reduced positioning accuracy [9]. Therefore, the mechanical conditions at the interface must be carefully analyzed, especially in systems with variable stiffness or time-varying external loads.

The main goal of this paper is to investigate the coupling and separation behavior in a nonlinear stiffness positioning stage that integrates a PZT actuator with a compliant amplification mechanism. This study focuses on the effects of preload, the influence of internal stress on actuator behavior and the derivation of a separation condition under dynamic excitation. The rest of this paper is organized as follows: Section 2 presents the system design and working principle. Section 3 provides the coupling analysis and separation criterion. Section 4 presents simulation and experimental results. Section 5 concludes the paper with key findings and suggestions for future work.

## 2. System Description

**Figure 1:**
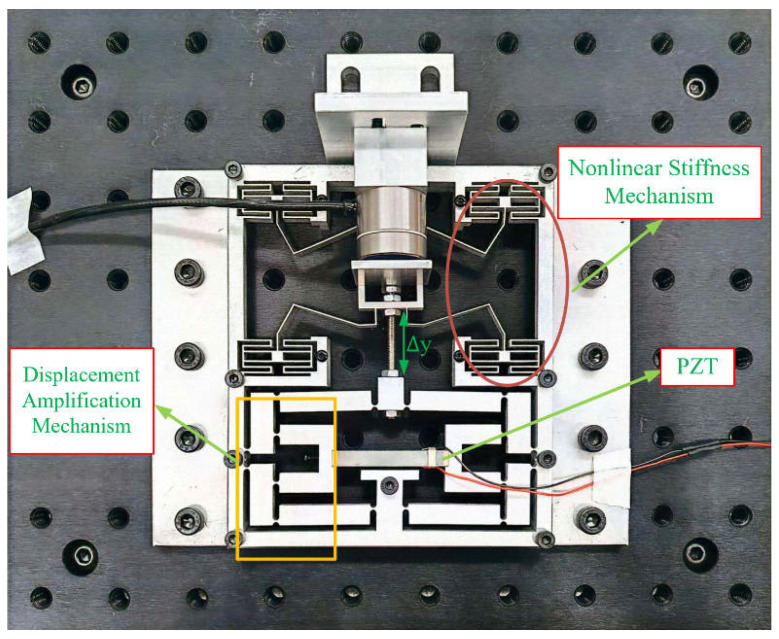
A developed compliant positioning stage actuated by a PZT actuator.

The developed precision positioning stage is shown in Figure 1, which combines a PZT with a compliant mechanism that allows both large-stroke motion and tunable stiffness [22]. The system is compromised of three main parts: a PZT actuator, a compliant displacement amplifier, and a nonlinear stiffness mechanism (NSM). These parts are assembly as one compact structure to ensure smooth and stable motion under different preload conditions.

The PZT is located at the center of the stage, serving as the main driving source. It is mounted on a rigid base and connected to the compliant amplification mechanism. This mechanism increases the small stroke of the PZT through a flexure structure that combines bridge-type and lever-type amplification, and it provides a displacement amplification ratio of 10.8. The stage is made from an aluminum plate using wire cutting, which ensures high precision and structural symmetry. The symmetric design helps guide the output motion in the correct direction and reduces undesired side movement [23,24,25].

The variable stiffness mechanism is located in the upper part of the platform. It consists of two symmetric compliant beams connected to a central preload component, which is adjusted by a bolt. Changing the position of the bolt alters the initial shape of the beams and affects their deformation behavior. This adjustment modifies the overall stiffness of the compliant structure. The method enables stiffness tuning without changing the structure or the material. The bolt applies a downward force on the beams, causing axial compression that increases stiffness through geometric effects. This adjustable stiffness helps maintain mechanical contact and ensures system stability under changing external forces.

The PZT is connected to the compliant amplification mechanism, which transfers the amplified motion to the output. The system employs two main sensors: a capacitive displacement sensor to measure the output stroke, and a digital torque screwdriver to measure the preload force. The displacement sensor is placed at the platform the NSM, aligned with the motion direction, to capture the total displacement. All sensor data are collected using a real-time myRIO-1900 (National Instruments, Austin, TX, USA) embedded system and processed through LabVIEW 2021 SP1 for synchronized data logging and control.

The PZT actuator is driven by a voltage amplifier, which receives control input signals from myRIO. The positioning stage is placed on a vibration isolated optical table to reduce external potential shock and vibrations. All components are fixed to a rigid baseplate using bolts to ensure stable mechanical grounding. The combination of adjustable stiffness and compliant amplification enables the platform to achieve precise positioning and adjustable dynamic performance within a compact design.

## 3. Piezoelectric-Mechanism Coupling Analysis

The integration of PZT actuator with a compliant mechanism offers a compact and high-resolution approach for precision motion systems. However, this combination leads to strong coupling effects. The compliant mechanism changes the mechanical boundary conditions of the actuator, which influences its dynamic behavior and output displacement. At the same time, the force and motion produced by the actuator affect the deformation and overall stability of the compliant mechanism. These effects are especially important when the system moves quickly or when a preload is added between the actuator and the structure.

A key challenge in these systems is the risk of separation between the actuator and the compliant mechanism. When the PZT contracts rapidly, low preload can cause detachment reducing motion transmission and causing instability. To avoid separation, preload is usually applied to maintain contact between components. However, preload also changes internal stress and may reduce the effective stroke of the actuator. To analyze this behavior, a dynamic model is needed to describe both the interaction between the actuator and the structure and the critical conditions that prevent separation.

In this study, several simplifying assumptions are adopted to establish the analytical model. First, the influence of parasitic motions is considered negligible due to their small magnitude compared with the primary displacement along the actuation direction. Second, the two contact interfaces are assumed to move symmetrically owing to the geometric symmetry of the compliant mechanism. Finally, the dynamic response is dominated by the first translational mode, which allows the system to be reduced to an equivalent single-DOF representation. These simplifications enable a tractable analytical model while capturing the dominant dynamic behavior of the actuator–mechanism system. Under this framework, a dynamic model is developed to describe the interaction between the actuator and the compliant structure [26]. The model is based on Lagrangian mechanics and uses a single input variable $u_{\mathrm{in}}$, which represents the displacement applied by the actuator.

The kinetic and potential energies of the actuator–mechanism system can be expressed in terms of the generalized coordinate $u_{in}$ as

(1) $$T=\frac{1}{2}M_{e}{\dot{u}}_{in}^{2},\;V=\frac{1}{2}K_{e}u_{in}^{2}$$

where $M_{e}$ and $K_{e}$ denote the equivalent mass and stiffness of the actuator–mechanism system, respectively. The motion of the system is governed by the following equation:

(2) $$\frac{d}{dt}\left(\frac{\partial T}{\partial {\dot{u}}_{\mathrm{in}}}\right)-\frac{\partial T}{\partial u_{\mathrm{in}}}+\frac{\partial V}{\partial u_{\mathrm{in}}}=F_{\mathrm{in}}$$

where $F_{\mathrm{in}}$ is the input force generated by the PZT.

To keep the actuator in contact with the compliant structure during fast motion, a separation condition is introduced. This condition is based on acceleration matching [19]. Let $a_{0}\left(t\right)$ and $a_{i}\left(t\right)$ be the absolute accelerations of the compliant structure and the actuator. At the instance of separation, these values must be equal:

(3) $$|a_{0}\left(t_{0}\right)\left|=\right|a_{i}\left(t_{0}\right)|$$

Assuming the system is excited with a harmonic signal, the acceleration values can be expressed using the excitation frequency and amplitude [27]. This leads to the following frequency-domain condition:

(4) $$f_{s}=\left(\sqrt{\frac{A_{0}+A_{i}}{A_{i}}}\right)f_{n}$$

where $f_{n}$ is the natural frequency of the compliant mechanism, $f_{s}$ is the minimum frequency required to maintain contact, and $A_{0}$, $A_{i}$ are the vibration amplitudes of the structure and the actuator, respectively. To avoid separation, the driving frequency $f_{d}$ must not exceed $f_{s}$. This leads to the stability condition:

(5) $$f_{s}\geq f_{d}$$

This condition describes the limits of actuator operation under dynamic loads, identifying the minimum preload and excitation frequency required to maintain contact and ensure consistent system performance.

### 3.1. Displacement Response Under Preload Conditions

**Figure 2:**
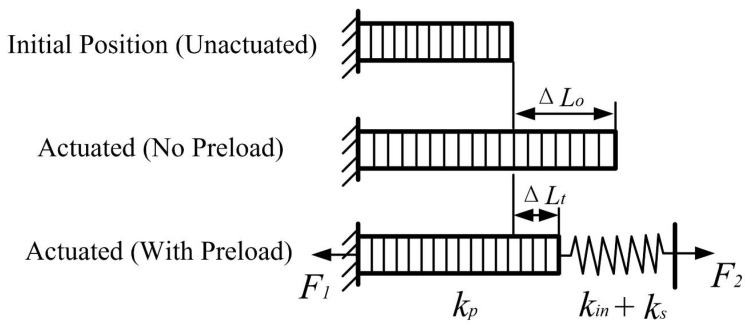
Output displacement of the PZT.

The coupling between the PZT and the compliant mechanism affects both the dynamic behavior and the output displacement of the actuator. As shown in Figure 2, a preload screw is used to preload the PZT to ensure firm contact with the compliant structure before actuation. This helps eliminate mechanical gaps, improve contact stability, and provide a consistent boundary condition for transmitting displacement.

However, due to the combined influence of preload and structural stiffness, the actuator may not reach its full free stroke. Instead, its effective displacement is reduced, which becomes critical in compact systems where stroke range is limited. This reduction can be quantitatively analyzed using static force balance at the interface between the actuator and the structure.

As illustrated in Figure 2, the actuator generates a driving force $F_{1}$ when a voltage is applied, which corresponds to a theoretical free stroke of $\Delta L_{0}$. However, when the actuator is connected to the compliant amplification mechanism and preload path, the actual displacement under load becomes $\Delta L_{t}$. Static equilibrium is established when the actuator force equals the total stiffness resistance, as given by

(6) $$F_{1}=K_{p}\left(\Delta L_{0}-\Delta L_{t}\right)$$

the external resisting force caused by the structural stiffness can be modeled as

(7) $$F_{2}=\left(K_{in}+K_{s}\right)\cdot \Delta L_{t}$$

here, $K_{p}$ is the stiffness of the PZT actuator, $K_{in}$ is the stiffness of the compliant mechanism, and $K_{s}$ represents the stiffness of the preload transmission path [28]. Under quasi-static conditions, assuming $F_{1}=F_{2}$, the force balance yields the following:

(8) $$K_{p}\left(\Delta L_{0}-\Delta L_{t}\right)=\left(K_{in}+K_{s}\right)\cdot \Delta L_{t}$$

solving Equation (8) for the loaded displacement $\Delta L_{t}$ gives

(9) $$\Delta L_{t}=\Delta L_{0}\cdot \frac{K_{p}}{K_{p}+K_{in}+K_{s}}$$

this expression quantitatively reveals the reduction in output displacement due to stiffness coupling and preload. When the total external stiffness $K_{in}+K_{s}$ increases, the output displacement becomes smaller. Therefore, both the stiffness and the preload should be carefully adjusted in the design.

To further incorporate the voltage-induced stroke and preload explicitly, the actuator force can also be modeled from an input–output perspective [29]. When preloads are applied, the PZT must first overcome the internal elastic forces of the surrounding structures before delivering motion. This effect can be modeled using force equilibrium at the actuator–structure interface as

(10) $$F=\left(C_{V}U_{\mathrm{in}}-\delta \right)K_{p}-\delta K_{\mathrm{in}}-F_{p}$$

where $F_{p}$ is the applied preload force and δ is the actual output displacement. Here, $x_{o}=C_{V}U_{in}$ represents the ideal stroke under voltage $U_{in}$, and the effective output $x_{1}$ is given by

(11) $$x_{1}=x_{0}\frac{K_{p}}{K_{p}+K_{in}+K_{s}}$$

This equation indicates that increasing the external stiffness leads to a reduction in output displacement. In the extreme case where the external stiffness greatly exceeds the actuator stiffness, the output is significantly diminished. This reduction arises from the combined effects of stiffness coupling and preload. The output displacement of actuator is inversely related to the total stiffness of the compliant mechanism and preload structure. As such, optimization of the preload is essential to maintain mechanical contact and minimize loss in performance.

### 3.2. Modal Analysis of the Positioning System

The natural frequency of a compliant mechanism is a key performance indicator in high-speed and precise positioning applications. It directly affects the system response speed and the positioning accuracy [30,31]. In integrated PZT-actuated platforms, the effective stiffness of the compliant mechanism is often influenced by preload, especially when the preload alters the internal stress distribution or the geometric profile of the flexure. To assess these effects, the positioning mechanism is typically modeled as a single-degree-of-freedom system and analyzed using an energy-based Lagrangian approach. The dynamic behavior is captured through equivalent stiffness *K* and equivalent mass *M*, which together define the dynamics of the structure [32]. The natural frequency of the mechanism can be estimated as

(12) $$\begin{array}{c} f_{n}=\frac{1}{2\pi }\sqrt{\frac{K}{M}} \end{array}$$

this relation provides a first-order approximation of the resonance frequency under small amplitude excitation.

For compound structures composed of multiple components such as flexures, actuators, and preload assemblies, the effective dynamic properties must be accurately combined. The effective mass $M_{e}$ of the system is obtained by summing the masses of all moving parts:

(13) $$M_{e}=M_{1}+M_{2}+M_{3}+\ldots +M_{n}$$

the effective stiffness $K_{e}$ depends on the connection topology of the substructures [33]. For serial configurations:

(14) $$\frac{1}{K_{e}}=\frac{1}{K_{1}}+\frac{1}{K_{2}}+\frac{1}{K_{3}}+\ldots +\frac{1}{K_{n}}$$

and for parallel connection:

(15) $$K_{e}=K_{1}+K_{2}+K_{3}+\ldots +K_{n}$$

substituting these into the frequency model yields the compound natural frequency:

(16) $$\begin{array}{c} f_{n}=\frac{1}{2\pi }\sqrt{\frac{K_{e}}{M_{e}}} \end{array}$$

this frequency is an important factor in defining the actuation range and the moment when separation may occur. A proper preload increases structural stiffness and improves dynamic performance. However, an excessive preload can create stress concentration or distort the geometry, which may result in over-stiffening and reduced motion amplification.

In variable stiffness mechanisms, deformation caused by preload influences the structural stiffness. This local change is represented by the tangent stiffness $K_{t}$, defined as

(17) $$K_{t}=\frac{dF(x)}{dx}$$

accordingly, the resonance frequency under a given preload force $F_{p}$ becomes

(18) $$f_{n}=\frac{1}{2\pi }\sqrt{\frac{K_{t}\left(F_{p}\right)}{M_{e}}}$$

In this study, the effective mass and stiffness are derived under the assumption of series-type behavior at the actuator–mechanism interface. For nonlinear responses, the system is linearized around the operating point and the tangent stiffness is employed to ensure that the frequency relation remains valid as a local approximation.

This formulation captures the effect of preload on dynamic behavior, because the tangent stiffness represents the local slope of the nonlinear force–displacement relationship and thereby reflects the nonlinear dependence of structural stiffness on preload. Consequently, properly tuned preload can expand the effective bandwidth and enhance responsiveness, while excessive preload may introduce adverse nonlinearities. A balanced preload is therefore essential to maintain both stability and dynamic performance.

### 3.3. Nonlinear Stiffness Effects Under Varying Preload

In variable stiffness mechanisms, a constant preload is typically applied during assembly to maintain reliable contact between the PZT and the compliant mechanism. This preload is generally adjusted by tightening a screw and remains unchanged during subsequent operation. However, the stiffness of the mechanism often changes during operation, such as repositioning an offset bolt. These changes affect the internal stress distribution and equilibrium configuration. As a result, the initially fixed preload may no longer match the current structural stiffness.

This mismatch becomes particularly critical when the system transits to a lower stiffness state. In such cases, the original preload sufficient under high-stiffness conditions may be inadequate to maintain contact, resulting in actuator structure separation. Conversely, if the preload is set to meet the lower stiffness requirement, the same preload may become excessive as stiffness increases, leading to unwanted compression of the PZT and reduced usable stroke.

**Figure 3:**
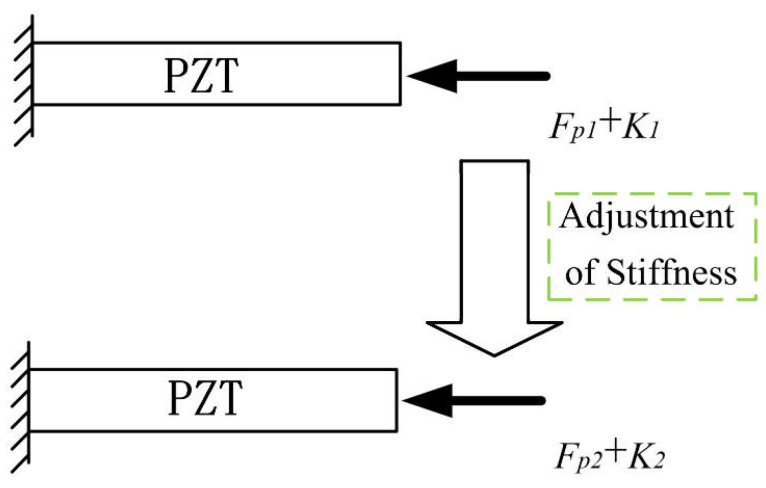
Dynamic change of preload force and stiffness in the tunable-stiffness mechanism.

Figure 3 illustrates this effect by showing how a constant preload $F_{p}$ when applied across varying stiffness configurations results in two different preload stiffness interactions, $F_{p1}+K_{1}$ in the high-stiffness state and $F_{p2}+K_{2}$ in the low-stiffness configuration, where $K_{1}$ and $K_{2}$ represent the variable stiffness of the compliant mechanism under different offset distance states.

When the stiffness decreases to a minimum value $K_{min}$, the fixed preload $F_{p}$ becomes inadequate, and additional actuator stroke is needed to compensate for the resulting gap. Such variations reduce the actuator stroke, decrease mechanical efficiency, and weaken the dynamic response, which may result in reduced reliability.

In summary, the mismatch between preload and variable stiffness creates a key limitation in systems that use a fixed preload. Without active adjustment, it is important to set the preload and stiffness properly during the design stage to ensure stable contact between the actuator and the structure under different working conditions.

## 4. Results and Experimental Validation

### 4.1. Results

#### 4.1.1. Static Displacement Response Under Preload

**Figure 4:**
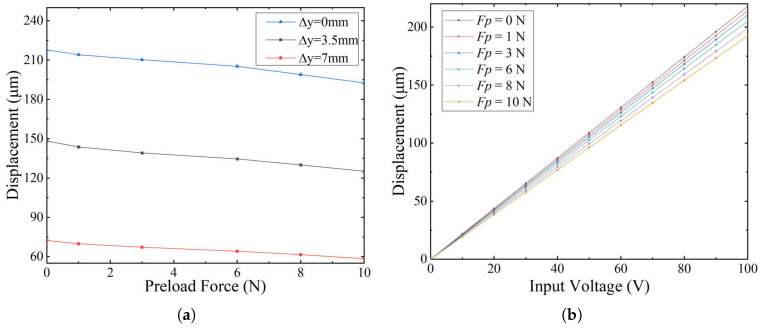
Static displacement performance under varying preload conditions. (**a**) Output displacement versus preload force at different offset distances (Δy=0, 3.5, 7 mm). (**b**) Voltage–displacement relation under different preload forces (Δy=0).

To investigate how preload force influences the static output behavior of the compliant positioning stage, a series of simulations were carried out under varying preload levels. The preload was applied between the PZT actuator and the compliant amplification mechanism, with the input voltage fixed at 100 V to ensure consistent excitation. As shown in Figure 4a, the output displacement decreases as the preload increases, regardless of the offset position. This demonstrates that preload significantly affects output displacement performance.

Specifically, Figure 4a presents the displacement–preload response for three offset distances: Δy=0, 3.5 mm, and 7 mm. For all cases, increasing the preload from 0 N to 10 N leads to a progressive reduction in maximum output displacement. The displacement at Δy=0 decreases from 216.73μm to 192.64μm, while at Δy=7 mm, it drops from approximately 71.81μm to 58.72μm. Although the absolute displacements differ due to geometric amplification effects, the decreasing trend remains consistent across all configurations. The influence of preload is more significant at smaller Δy values, emphasizing the role of initial alignment in amplification performance.

To further assess the consistency of actuation behavior under varying geometric conditions, Figure 4b presents the displacement response as a function of input voltage at a fixed offset distance of Δy=0. Although the absolute displacement differs across various Δy values, simulations show that the voltage–displacement relationship exhibits a linear trend under all tested preload levels. Therefore, the case of Δy=0 is chosen as a reference to illustrate the characteristic behavior. The results indicate that although preload reduces the displacement amplitude, the actuator preserves stable and consistent behavior across the input range. This confirms that a moderate preload is effective for maintaining contact stability and motion precision.

Overall, the results confirm a trade-off between contact reliability and output amplitude. While preload is required to maintain contact, excessive preload increases the system stiffness, limiting displacement. An appropriate preload ensures mechanical stability and efficient motion output.

#### 4.1.2. Dynamic Response Under Preload

To evaluate the dynamic performance of the compliant positioning stage, both modal and transient simulations were performed under different preload conditions. Modal analysis was conducted to extract the first natural frequency (FNF), which served as an indicator of system stiffness. Transient simulations were performed to evaluate the contact continuity between the actuator and compliant mechanism under periodic excitation.

**Figure 5:**
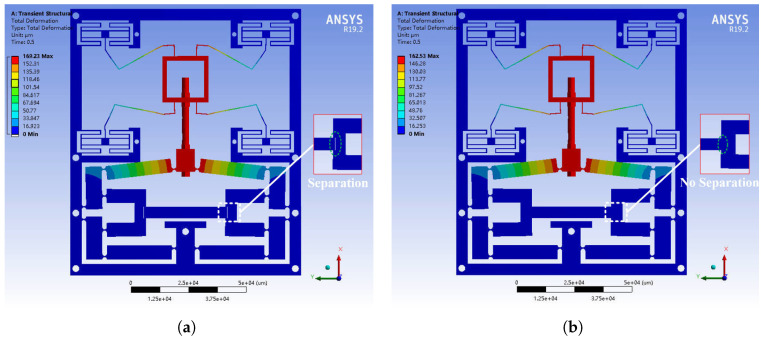
Dynamic response under preload conditions. (**a**) Contact separation under insufficient preload. (**b**) No contact separation under sufficient preload.

**Figure 6:**
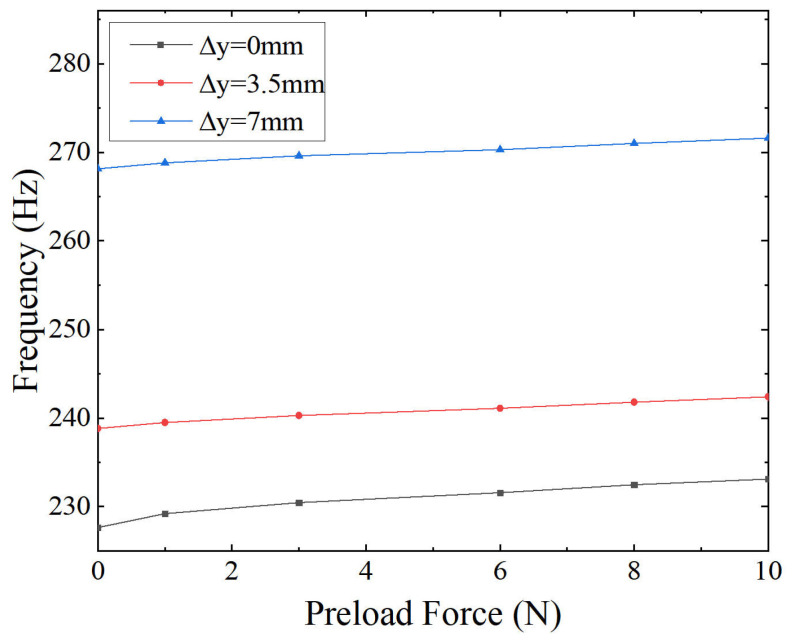
Variation of the FNF under different offset distances.

The results are presented in Figure 5 and Figure 6. As shown in Figure 5a, contact separation occurred when the preload was insufficient. In contrast, Figure 5b illustrates that applying adequate preload ensured continuous contact during the motion. Furthermore, Figure 6 presents the variation of FNF with respect to preload level. An increasing trend is observed, indicating that higher preload leads to greater system stiffness.

The results indicate that the offset distance (Δy) plays a dominant role in determining the dynamic stiffness. Configurations with a larger Δy result in higher initial FNF values due to changes in internal stress and geometric nonlinearity in the flexure elements. In comparison, preload plays a secondary role by improving axial compression and enhancing mechanical contact.

Figure 6 shows that FNF increases with preload across all offset configurations. However, the rate of increase is nonlinear. At lower preload levels, the frequency increases rapidly due to improved axial contact and reduced micro-separation. As the preload further increases, the frequency growth rate gradually decreases, indicating a diminishing contribution of additional compression to dynamic stiffness. This saturation behavior reflects a reduced stiffening effect of preload under higher compression, particularly in geometrically constrained configurations.

From the simulated transient results, Figure 5a shows that low preload leads to contact loss and delayed mechanical response, causing unstable dynamics. In contrast, Figure 5b, shows that sufficient preload maintains full contact during motion, resulting in consistent displacement and suppression of residual vibrations.

These results show that offset distance plays a key role in enabling nonlinear stiffness variation. In the meanwhile, preload also affects the overall stiffness and nonlinear behavior of the mechanism. Therefore, both geometric configuration and preload should be considered as a whole in the design of compliant stages to achieve stable and tunable dynamic performance.

#### 4.1.3. Validation of the Separation–Avoidance Criterion

To maintain stable contact between the PZT and the compliant structure during motion, the separation–avoidance criterion given in Equations (4) and (5) is applied. This criterion provides a theoretical lower limit for the required preload displacement to avoid detachment during actuation. It indicates that the preload displacement $A_{0}$ should be larger than the driving amplitude $A_{i}$.

In this study, the natural frequency of the compliant structure was identified as 227.64Hz through modal analysis, while the driving frequency was set to $f_{d}=500\;\mathrm{Hz}$ to represent an excitation condition [22]. The actuation amplitude was defined as $A_{i}=10\;\mu m$, corresponding to the nominal stroke range of the PZT. Based on these values and Equation (5), the analytical inequality suggests that the minimum required preload force is approximately 7.31 N.

**Figure 7:**
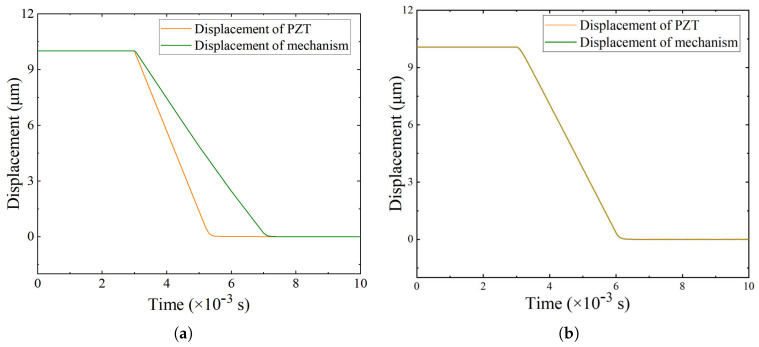
Displacement response of the stage and PZT. (**a**) With separation. (**b**) Without separation.

To verify this theoretical prediction, transient simulations were performed under different preload force. As shown in Figure 7a, when the preload is smaller than the theoretical value, separation occurs between the PZT and the compliant mechanism during motion. In contrast, Figure 7b shows that with sufficient preload, the PZT stays in contact with the compliant mechanism during the entire motion, and no separation occurs.

The simulation results validate the analytical criterion across different preload conditions. When the preload is lower than the theoretical requirement, separation occurs because the actuator motion exceeds the recovery capacity of the compliant mechanism. Once the preload surpasses the theoretical requirement, contact is maintained during the entire motion. This confirms that the criterion effectively predicts the preload needed to prevent detachment under dynamic excitation and reflects the combined effect of actuation amplitude, frequency, and structural stiffness.

### 4.2. Experimental Validation

This section provides experimental tests of the mechanism under different conditions. The aim is to evaluate the output performance of the actuator under varying preload and offset distance. The results are also used to verify the dynamic response of the compliant mechanism.

#### 4.2.1. Experimental Results

To investigate the effect of preload on the static displacement response of the compliant stage, displacement and voltage measurements were carried out using a capacitive displacement sensor. The actuator was driven by a stepwise voltage input ranging from 0 to 100 V. Three offset distance conditions were tested, corresponding to Δy values of 0 mm, 3.5 mm, and 7 mm. For each configuration, preload values of 0 N and 10 N were applied by adjusting the compression using mechanical screws.

**Figure 8:**
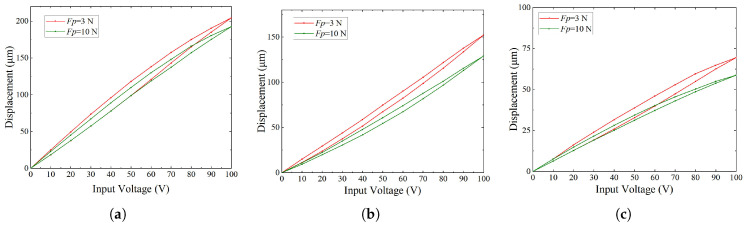
Output displacement of stage. (**a**) Offset distance Δy=0 mm. (**b**) Offset distance Δy=3.5 mm. (**c**) Offset distance Δy=7 mm.

As shown in Figure 8a, when Δy is 0 mm, increasing the preload from 0 N to 10 N reduces the maximum displacement from 212.35μm to 189.33μm. At Δy=3.5 mm, the displacement decreases from 152.1μm to 129.42μm, as shown in Figure 8b. For Δy=7 mm, it further drops from 69.34μm to 54.35μm. These results indicate that preload consistently reduces output displacement across different offset distance, and the reduction becomes more pronounced as the offset increases.

Additionally, the curves in Figure 8 show that preload reduces the displacement amplitude in all cases, with stronger effects when the offset distance is larger. The change in slope between 0 N and 10 N reflects the influence of preload on stiffness. This effect becomes more evident in the configuration with Δy equal to 7 mm, where geometric nonlinearity increases the sensitivity of stiffness to preload.

Moreover, a hysteresis loop can be observed in Figure 8, which is a typical nonlinear effect of PZT actuators. This phenomenon may introduce positioning errors in open-loop operation [34,35,36]. To mitigate this, closed-loop control will be considered in future work to reduce hysteresis-induced errors and improve positioning accuracy.

**Figure 9:**
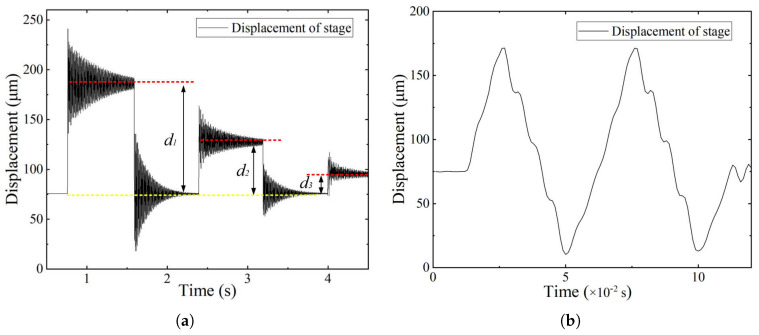
Output displacement of the stage under insufficient preload causing sepration. (**a**) Square excitations. (**b**) Triangular excitations.

Dynamic tests were conducted to examine the contact behavior of the system under periodic excitation. A voltage of 100 V was used to drive the actuator, and the output displacement was acquired under preload force. As shown in Figure 9, the response to square excitation (Figure 9a) exhibits amplitude decay over time ($d_{1}$ > $d_{2}$ > $d_{3}$), along with discontinuous transitions and high-frequency oscillation. The red line denotes the output displacement level, whereas the yellow line denotes the baseline displacement level. These features indicate repeated contact loss and unstable motion.

In contrast, the response to triangular excitation (Figure 9b) shows a different separation pattern. During the initial rising phase (0–0.025 s), the PZT and compliant mechanism remain in contact. However, during the falling phase (0.025–0.05 s), the displacement becomes nonlinear and asymmetric, indicating that separation occurs as the PZT retracts.

**Figure 10:**
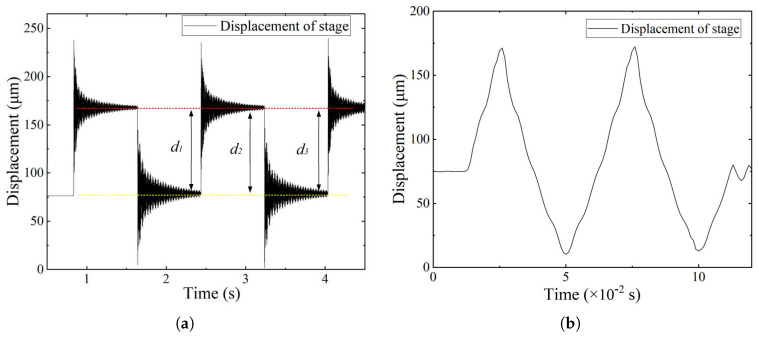
Output displacement of the stage under sufficient preload ensuring no-separation. (**a**) Square excitations. (**b**) Triangular excitations.

Figure 10 presents the output displacement of the stage under no-separation conditions with two types of periodic excitations. In Figure 10a, the response to square wave excitation is stable, with consistent displacement amplitudes and smooth transitions at each voltage step. No sudden displacement changes or rapid vibrations indicate continuous contact between the PZT and the compliant mechanism during the entire motion. In Figure 10b, the displacement under triangular excitation exhibits a symmetric waveform with regular rise and fall transitions. The consistent shape and amplitude of the response confirm that the actuator and the mechanism remain mechanically coupled during the entire motion.

**Figure 11:**
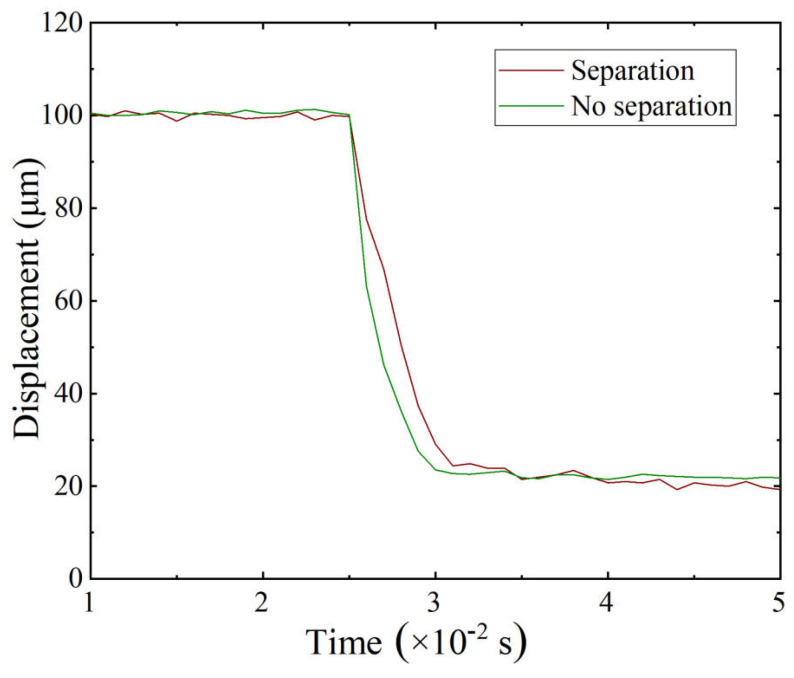
Displacement of the stage with and without separation.

To further confirm the influence of contact conditions, Figure 11 compares the transient displacement responses under separation and no-separation cases. The no-separation shows a stable state, while the separation case results in smaller displacement and residual vibration. These results demonstrate that the proposed preload condition maintains reliable mechanical contact.

**Figure 12:**
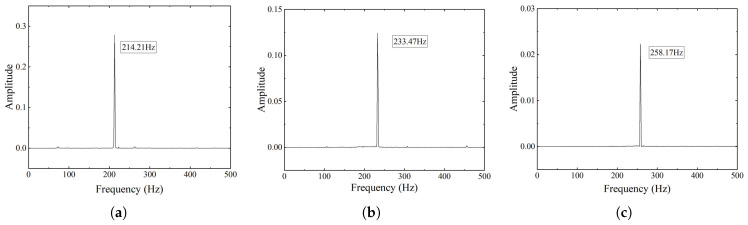
Experimental results for the FNF of the stage under a preload force of 10 N. (**a**) Δy=0 mm. (**b**) Δy=3.5 mm. (**c**) Δy=7 mm.

Frequency response tests were conducted to examine the effect of preload force on the resonant behavior of the system. These tests were designed on the theoretical basis of Equations (17) and (18), which relate the tangent stiffness to the preload-dependent resonance frequency. The measured FNFs at various offset distances are presented in Figure 12. Under a constant preload of 10 N, the measured FNF increases with offset distance. Specifically, the measured values are 214.21 Hz at Δy=0 mm, 233.47 Hz at Δy=3.5 mm, and 258.17 Hz at Δy=7 mm. These values are significantly higher than those under low-preload conditions. This result shows that preload increases the resonant frequency of the system. This experimental result is consistent with the simulation results shown in Figure 6, with a maximum error of less than 8.8%. Compared to the low-preload condition, applying a high preload increases the modal frequency and improves the overall stiffness of the system.

#### 4.2.2. Discussion

The experimental results support the theoretical predictions that both preload and offset distance affect the mechanical response of the actuator. Nonlinear stiffness adjustment is determined by offset distance, while preload affects displacement amplitude and contact stability. Moreover, frequency response analysis shows that increasing preload leads to a gradual increase in resonance frequency and follows a nonlinear growth trend. A reasonable preload enables the system to maintain stable contact and excessive preload reduces the output displacement of the stage.

The results confirm the accuracy of the proposed models in describing system behavior. Both the geometric configuration and preload adjustment significantly influence performance. The offset distance determines the extent of stiffness adjustment and the resulting displacement output. Preload enhances stability and prevents separation during operation.

## 5. Conclusions

This study presents the design and experimental validation of a compliant positioning stage with tunable nonlinear stiffness achieved through geometric offset and preload adjustment. The results confirm that the offset Δy is the dominant factor in achieving stiffness adjustment, while the preload primarily improves contact stability and dynamic robustness. The theoretical model accurately predicts displacement and frequency variations, consistent with simulation and experimental data. Static and dynamic tests show stable displacement responses to voltage under preload and indicate that the appropriate preload effectively prevents separation during high-frequency actuation. These findings provide a foundation for future development of multi-axis compliant systems that integrate sensing and adaptive control to enhance precision and functional flexibility.

## Data Availability

The original contributions presented in the study are included in the article, further inquiries can be directed to the corresponding author.

## Abbreviations

The following abbreviations are used in this manuscript:

PZT	Piezoelectric actuator
FNF	First natural frequency
NSM	Nonlinear Stiffness Mechanism
